# Ayurvedic Response to COVID-19 Pandemic in Kerala, India and Its Impact on Quarantined Individuals – A Community Case Study

**DOI:** 10.3389/fpubh.2021.732523

**Published:** 2021-10-15

**Authors:** Sharmila Mary Joseph, Divya S. Iyer, Rajmohan Velayudhan Pillai

**Affiliations:** ^1^Department of Ayush, Government of Kerala, Thiruvananthapuram, India; ^2^Government Ayurveda Medical College, Thiruvananthapuram, India

**Keywords:** Ayurveda, Kerala, SARS-CoV-2, COVID-19, implementation, decentralization, Amritham, traditional medicine

## Abstract

The SARS-CoV-2 infection has resulted in massive loss of valuable human lives, extensive destruction of livelihoods and financial crisis of unprecedented levels across the globe. Kerala, a province in India, like the rest of the country, launched preventive and control measures to mitigate the impact of COVID-19 early in 2020. The Government of Kerala started 1206 Ayur Raksha Clinics and associated Task Forces across the state in April 2020 to improve the reach and penetration of Ayurvedic preventive, therapeutic and convalescent care strategies for the COVID-19 pandemic. The implementation framework of the strategy was properly designed, and had a decentralized, people-centered, and participatory approach. Kerala has robust public health machinery with adequate human resource and infrastructure in the conventional medicine sector. This community case study examines how the decentralized organizational framework was effectively utilized for facilitating the delivery of Ayurvedic services in the COVID-19 situation. Key observations from the study are: Ayurvedic programs implemented systematically, under an organized framework with social participation enables wider utilization of the services. Such a framework is easily replicable even in resource-poor settings. Rather than a pluralistic approach, an integrative health system approach may be more viable in the Kerala scenario in public health emergencies.

## Introduction

The World Health Organization declared the COVID-19 outbreak as a global pandemic in March 2020 (1). The novel coronavirus has wreaked havoc across the globe, adversely impacting the health of the people and the public health machinery and disrupting economies world-wide. Most countries launched mitigatory steps to check the spread of infection, which included multisectoral emergency responses, establishment of quarantine or isolation centers, setting up of diagnostic and treatment facilities, and adequate intensive care services (2). A definite antiviral treatment protocol for SARS-CoV-2 is yet to be firmly established (3). However, clinical management protocols with repurposed antivirals, steroids and immunomodulators have demonstrated significant results (4–8). As of 28 June 2021, the number of human lives lost in this pandemic is over 39 million (9). Although the destruction of livelihoods from trade and financial collapse has remained detrimental, particularly in resource-poor countries, the loss of valuable human lives worldwide has burdened the rich and poor alike (10–13). With more than 30 million cases in India as of 28 June 2021 and with danger of a third wave looming large over the country, the federal (central) and provincial (state) governments have taken concerted efforts to accelerate the vaccination drive, coupled with active management of all positive cases and coordinated preventive and mitigatory strategies (14, 15).

The traditional medicine systems across the globe, based on the available practices and knowledge, designed protocols for the prevention and management of early COVID-19 (16–20). The Ministry of Ayush, Government of India (Ministry handling Ayurveda, Yoga and Naturopathy, Unani, Siddha and Homeopathy) recommended various prophylactic measures based on Ayush systems against the pandemic as early as 20 March 2020 (21). Further, the Ministry in November 2020 released the National Clinical Management Protocol for Ayurveda and Yoga, based on knowledge from Ayurveda treatises, empirical evidence, experience from clinical practice and preliminary results from ongoing clinical studies (22). Considerable data regarding the utilization of traditional medicines against the COVID-19 pandemic have also been emanating worldwide (23).

Kerala is a small state in the southern part of India with a dense population of 34 million (24). Despite a low per capita income, Kerala has achieved high social and human development levels, with remarkable outcomes in health, poverty reduction, affordable basic education facilities and high literacy levels (25, 26). Some of the social development achievements of Kerala are comparable with those of developed nations, so much so that the social development story of the state is christened ‘The Kerala Model' (27). The core of the model is the people-centered, decentralized, social and participatory governance system focusing on delivery of basic health and education services with the active collaboration of local-self-governments under the state government (28).

Ever since the first Indian case of the SARS-CoV-2 infection was reported from Kerala, the state government was on alert to handle any emerging crisis. With a significant influx of COVID-19 positive persons from other states and countries in the early phases of the pandemic, Kerala built up an active COVID-19 containment strategy (29). The state also stepped-up contact tracing, testing and tracking in a bid to flatten the curve and to minimize the burden on hospitals (30). The state health machinery strongly emphasized the need for Covid-appropriate behavior viz. social distancing, masking, and proper hand sanitizing. The protocol followed in Kerala is modelled on the guidelines issued by the federal government and the WHO protocols. These guidelines are fine-tuned regularly based on inputs from study reports and research findings. In addition to the accepted pharmacological and non-pharmacological interventions, traditional medicines (Ayurvedic medicines) are also being used to boost the innate immunity of individuals and to manage early uncomplicated cases of COVID-19 infection, as adjuvant to conventional management protocols (31). Ayurvedic management for infectious diseases in Kerala is generally well-accepted and provided through public and private Ayurvedic dispensaries and hospitals. However, the therapeutic results of Ayurvedic management protocols have not been systematically documented.

Kerala's robust COVID-19 containment strategy yielded good results right from the early days of the pandemic, as evident from published data. The seven-day average of the newly reported cases and deaths in Kerala as of 31 July 2020 was 945 and three respectively (29, 32, 33). Subsequently, evident community transmission surfaced, with newly reported cases and deaths reaching over 8,700 and 23 as of 15 October 2020. As of 8 May 2021, at the peak of the second wave of the pandemic, the daily caseload rose to over 40,000 with a case fatality rate (CFR) of 0.3% (CFR in India was 1.9% in this period) (34). Kerala has now launched the ‘Back to the Basics' policy placing added emphasis on mitigation measures (35).

Kerala has a vibrant public health machinery in the conventional medicine sector with over 50,000 beds in 1284 healthcare facilities. The state has also deployed over 6,000 doctors and approximately 21,000 paramedical staff and other skilled and unskilled workers under the above machinery to implement and report various healthcare activities (36). Further, direct social participation in community health is active in Kerala, resulting from the effective integration of the public health system with the local self-government systems (37). However, in comparison with the conventional health system, the Ayurveda Department does not have as many resources, either on the manpower front or the infrastructure front. There are 947 public Ayurvedic health care facilities in Kerala managed by about 1,500 Ayurvedic doctors. Inpatient services are provided through 130 public Ayurvedic hospitals, with a bed strength of 3,154. On account of these constraints, the service delivery system in the Ayurveda side of public health was not as extensive as the general health system (36).

In the early phase of the pandemic, Government of Kerala (GOK) constituted a seven-member Task Force to prepare the blue-print of an Ayurvedic strategy for prevention and mitigation of COVID-19 (38, 39). GOK thereafter approved a comprehensive organizational algorithm and an action plan for implementing the strategy. The Ayush Department structured the entire implementation framework on a participatory model, with active involvement of various Ayurvedic professional organizations and local self-governments (LSGs). An essential drug list was also approved for prevention and mitigation of COVID-19, after collating opinions of over 300 Ayurvedic experts and drawing inputs from the guidelines of the Health Department, GOK and Ayush Ministry, Government of India (40).

This community case study examines how a policy-oriented, decentralized, people-centered and participatory framework helped effective service delivery through Ayurveda and facilitated systematic reporting during the initial wave of the pandemic in Kerala. The paper also observes the impact of such an organized approach in the coverage of the Ayurvedic preventive program–Amritham and its preliminary effect on COVID-19 quarantined individuals in Kerala from 21 May 2020 to 8 July 2020.

## Materials and Methods

The community case study looks into a pre-designed Ayurvedic strategy comprising a well-defined organizational framework to execute diverse Ayurvedic programs for the COVID-19 pandemic in Kerala. The authors were responsible for successfully operationalizing these programs and acquired first-hand experience and access to primary data of Ayurvedic Covid programs.

### Key Stakeholders

The key stakeholder is the GOK represented by the Department of Ayurveda, Yoga and Naturopathy, Unani, Siddha and Homeopathy (Ayush) with the main players being Department of Ayurveda Medical Education, Department of Indian Systems of Medicine, and the National Ayush Mission (State Mission Office). The State Ayurveda COVID-19 Response Cell (SACRC) coordinated activities of various departments, and LSGs for implementing the approved programs.

### The Implementation Framework

**Figure 1:**
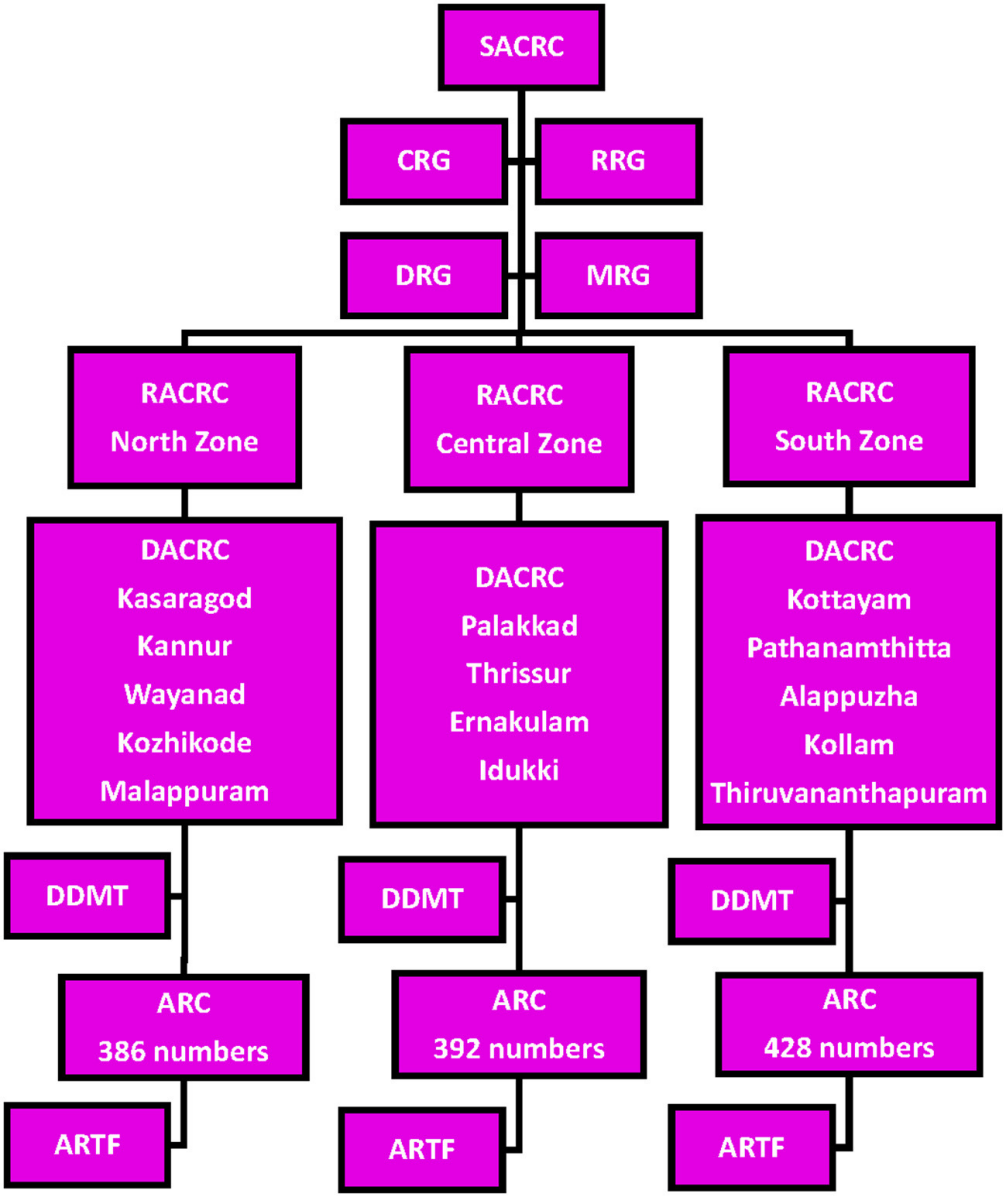
The organizational algorithm of Ayurvedic Response in Kerala. SACRC, State Ayurveda COVID-19 Response Cell; RACRC, Regional Ayurveda COVID-19 Response Cell (based on three zones of the state); DACRC, District Ayurveda COVID-19 Response Cell (function at the 14 districts of Kerala); ARC, Ayur Raksha Clinics (work at village level); ARTF, Ayur Raksha Task Force (ground level supporting body of ARC); CRG, Clinical Resource Group; RRG, Research Resource Group; DRG, Drug Resource Group; MRG, Multimedia Resource Group; DDMT, District Data Management Team.

The implementation framework broadly encompassed Ayurveda COVID-19 Response Cells at various levels with the support and participation of the key stakeholders. The SACRC was the coordinating body entrusted with the responsibility to design, implement, evaluate and report different Covid related activities (39). The state cell was also responsible for preparing guidelines and coordinating downstream controlling bodies and various stakeholders to implement the approved programs. In order to support SACRC to devise guidelines and advisories based on clinical aspects of the pandemic, to ensure systematic collection and management of data regarding implementation, community-level mass drug administration and to generate public awareness through different media platforms, the Ayush department constituted the clinical, research, drug and multimedia resource groups respectively. Apart from regional coordination of activities, Regional Ayurveda COVID-19 Response cells gave academic inputs and human resource support to the district response cells. The regional cells functioned from three Government Ayurveda Medical Colleges situated at Thiruvananthapuram, Ernakulam and Kannur districts representing the southern, central and northern regions of the state. The 14 District Ayurveda COVID-19 Response cells were responsible for delivering the Ayurvedic services to the Kerala population. The district cells were in charge of the Ayur Raksha Clinics (which means ‘life protection clinics' or simply ‘Life Clinics') across the state, the basic units for implementing the Covid mitigation programs. These clinics functioned at all government Ayurveda institutions in the primary, secondary and tertiary levels viz. Ayurveda dispensaries, Ayurveda hospitals and Ayurveda Medical Colleges with broad support from LSG bodies. Figure 1 depicts a schematic representation of the implementation framework. The execution of various programs was principally government-funded with additional support from LSGs.

### Social Participation

The government operationalized the Ayur Raksha Clinics (ARC) and the Ayur Raksha Task Force (ARTF) as early as mid-April 2020. The ARTF included Ayurveda Medical Officer of the concerned ARC, the designated officials from the LSGs, Accredited Social Health Activists (ASHA), local health volunteers, private Ayurvedic practitioners and students, faculty and other staff of Ayurveda Medical Colleges and the elected representatives from the community. The ARTF was responsible for delivery of Ayurvedic medicines and services, follow-up, data collection and reporting.

### Various Ayurvedic Programs for the COVID-19 Pandemic

From the onset, the GOK implemented various Ayurvedic Preventive Strategies (APS) to strengthen the innate immunity of the public against COVID-19. The strategy covered different categories of the population based on the age and risk of exposure. These were Swasthyam (which means good health) for general population below 60 years, Sukhayushyam (which means heathy old age) for general population above 60 years, and Amritham (which means nectar) for the Covid quarantined population. The strategy of Punarjani (which means rejuvenation) intended to ensure a speedy return to normal health in individuals in the post-Covid phase without any lingering Covid sequelae. Further, the government implemented the Bheshajam (which means free from disease fear) program, providing Ayurvedic treatments to asymptomatic or mildly symptomatic COVID-19 patients who had given consent for the same. Table 1 provides details of various Ayurvedic programs.

**Table 1:** Details of various Ayurvedic programs for COVID-19 in Kerala as of 28 June 2021.

**Program**	**Details**	**Policy document from Government of Kerala**	**Number of beneficiaries**
Swasthyam	APS^*^ for general population below 60 years	G.O. (Rt) No.156/2020/AYUSH Dated, Thiruvananthapuram, 8 April 2020	1,059,235
Sukhayushyam	APS for general population above 60 years	G.O. (Rt) No.156/2020/AYUSH Dated, Thiruvananthapuram, 8 April 2020	673,727
Amritham	APS for COVID-19 quarantined individuals	G.O. (Rt) No.180/2020/AYUSH; dated, Thiruvananthapuram, 15 May 2020	809,756
Bheshajam	ATS^#^ for uncomplicated COVID-19 cases (Category A)	G.O (Rt) No. 425/2020/AYUSH dated, Thiruvananthapuram, 18 November 2020	287,093
Punarjani	ATS for covid convalescent care	G.O. (Rt) No.156/2020/AYUSH Dated, Thiruvananthapuram, 8 April 2020	361,551

* *Ayurvedic Prophylactic Strategies*,

# *Ayurvedic Treatment Strategies*.

The state cell circulated specific Standard Operating Procedures for implementation of the above-mentioned programs. The life clinics and task forces actively engaged with local social media groups and propagated Ayurvedic tips on daily living activities and diet. The Ayurvedic COVID-19 response cells also maintained social media platforms for propagating the Ayurvedic programs on healthy lifestyles and dietary tips. Oushadhi (which means medicinal herbs), the Pharmaceutical Corporation Kerala Limited, a Good Manufacturing Practice (GMP) certified company under the Government of Kerala manufactured and supplied the Ayurvedic medicines used for the above programs.

### Data Collection and Reporting

The State Ayurveda COVID-19 Response Cell organized training programs on data collection, processing and management and various epidemiological methods for the district data management team and Ayurvedic physicians in charge of the life clinics. It also delivered necessary instructions as video tutorials. The life clinics and their task forces collected primary data from the targeted populations. They reported data to the district Covid cells, where the data management team verified and subsequently shared it with the state cell to evaluate and generate consolidated reports with recommendations.

### Observations on Amritham – The Ayurveda Supported Quarantine Care Program

We conducted a preliminary analysis of the data in respect of the Ayurvedic quarantine care offered through life clinics across the state Amritham program). The analysis assessed the coverage of the program among the COVID-19 quarantined individuals in Kerala from 21 May 2020 to 8 July 2020 when delivered through the approved implementation framework. We also conducted a preliminary analysis of the preventive strategies on the quarantined individuals during the same period. The study considered the comprehensive data regarding the relevant sociodemographic status, comorbidity status, and quarantine course of all the individuals who registered under Amritham and the number of quarantined individuals who tested positive for SARS-CoV-2 infection from the program. The total number of quarantined individuals and the number of individuals who tested positive for SARS-CoV-2 virus was collected directly from the local health authorities by the district data management team and verified against data from authentic government sources, and the same was confirmed and compiled at the state level (33, 41).

### Quarantine Strategy in Amritham

During the study period, the declared quarantine duration for an individual who travelled to Kerala or for a primary or secondary contact of a SARS-CoV-2 infected person was 14 days (42). Any person travelling to the state had to register on an official web portal so that the government mechanism could ensure that these individuals quarantined themselves either in their homes or in designated quarantine institutions (43). If the Rapid Response Team (a team of volunteers from the locality under the local self-government) at the individual's destination found the quarantine facilities at their homes to be inadequate, or unsuitable for an isolated stay due to the presence of aged cohabitants or lack of facilities, the local health authorities advised institutional quarantine. The local authorities shared the information of quarantined individuals in the locality with the life clinics on a daily basis, and the clinics contacted all quarantined individuals and enrolled those who were willing to adhere to the protocols prescribed under Amritham.

### Ayurvedic Preventive Strategies for COVID-19 Under Amritham

The APS included polyherbal Ayurvedic formulations in varying dosage forms, including decoctions, tablets, pills, and powders, for internal use and as herbal fumigations (40). The Ayurvedic physicians at the life clinics also had the liberty to give personalized preventive medicines from the essential drug list, for various age groups, after considering diverse Ayurvedic protocols like body constitution (Prakriti) of the quarantined individuals and their associated comorbidities (44). The District Ayurveda COVID-19 Response cells constituted medical advisory boards in all districts to monitor the prophylactic and therapeutic interventions delivered under various approved programs.

### Data on Amritham

The life clinics and task forces were responsible for delivering Ayurvedic medicines to the individuals under quarantine and for collecting data regarding the compliance of quarantined individual, the individual's health status, and the development of any Covid symptoms during the quarantine course. They obtained informed consent for consumption of Ayurvedic medicines from the quarantined individuals and gave personalized instructions regarding the use of Ayurvedic medicines, dietary advice, and lifestyle modifications and did tele-follow-up. The life clinics adhered to specific criteria for reporting the successful completion of quarantine under Amritham by an individual to the state cell, as follows;

1. The individuals should have started Ayurvedic medicines within three days of the commencement of their quarantine period.
2. The quarantined individuals should have consumed the APS medicines continuously for a minimum period of three days before undergoing tests for detecting SARS-CoV-2 infection.
3. The quarantined individuals should not have been under any prophylactic cover against SARS-CoV-2 infection with medicines from other medical disciplines or Ayurvedic medicines not recommended as per the essential drug list.
4. The quarantined individuals should not have any serious comorbidities or should not be debilitated.

## Results

**Figure 2:**
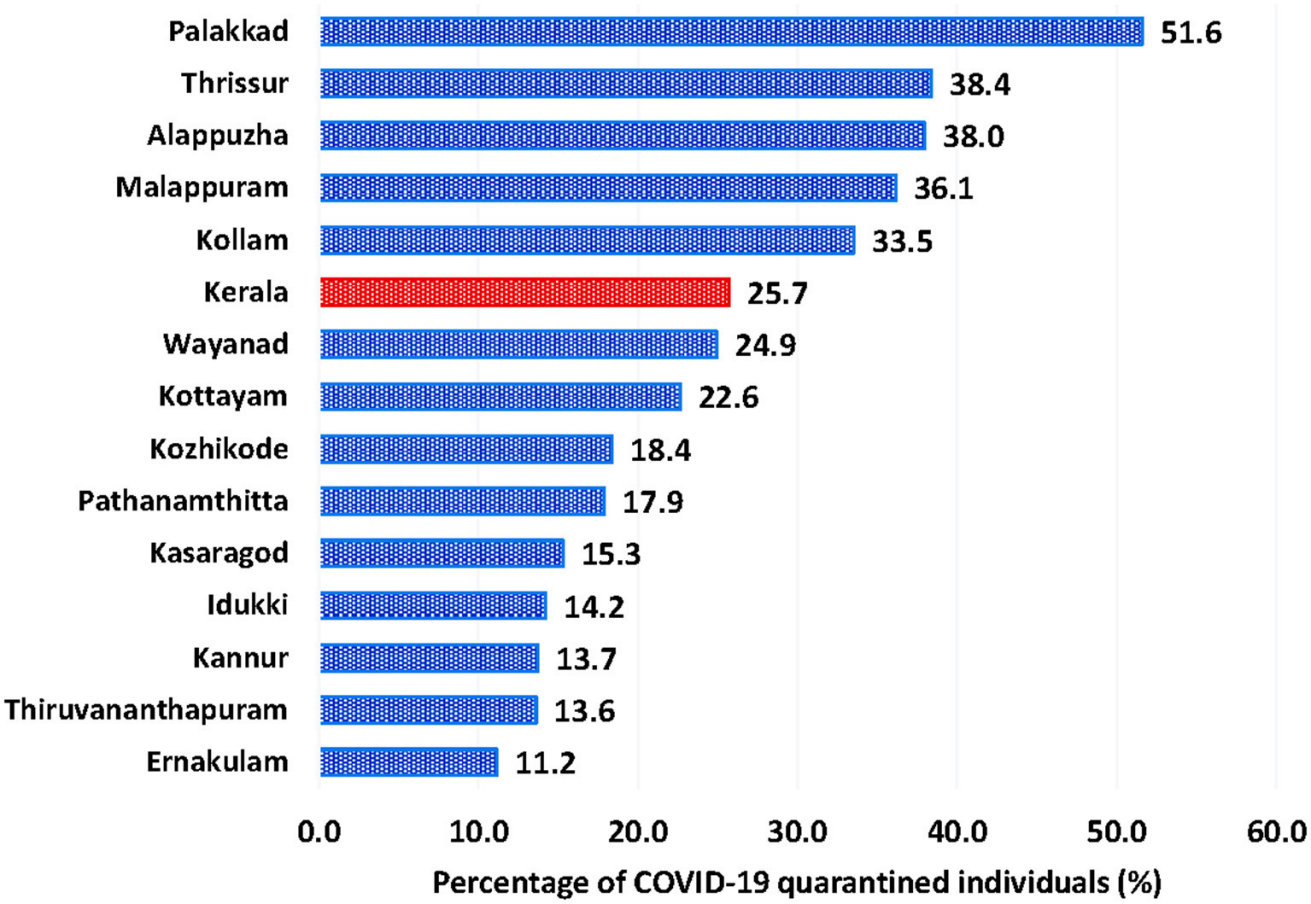
State-wide and district-wise percentage of COVID-19 quarantined individuals enrolled in Amritham from 21May 2020 to 8 July 2020.

Table 1 shows the data regarding the reach of various approved Ayurvedic programs operationalized through the implementation framework. The Ayush Department introduced 1,206 life clinics and associated Task Forces across the state covering all villages, to improve the reach and penetration of Ayurvedic preventive, therapeutic and convalescent care strategies for the COVID-19 pandemic. Of the 394,269 individuals quarantined in the state from 21 May 2020 to 8 July 2020, 101,218 (25.7%) individuals were covered under Amritham (Figure 2). Out of the 27,554 quarantined individuals in Palakkad district (one of the 14 revenue districts in Kerala) during the same period, 51.6% received Ayurvedic preventive medicines under Amritham, which was the highest in the state. Thrissur with 38.4%, Alappuzha with 38%, Malappuram with 36.1% and Kollam with 33.5% coverage for the program were the other leading districts (Supplemental Tables 1, 2). Among the quarantined individuals under Amritham care, 80% belonged to the age group of 15 to 54 (Table 2), 73% were males, and 97.4% had established travel history, with 63.1% constituting re-immigrants. The majority (92.2%) of the 101,218 individuals under Amritham were quarantined at their own homes and remaining at various institutional facilities (Table 3). Out of the individuals quarantined under the Amritham program, 19.2% reported comorbidities, 72.5% of whom were males. Diabetes mellitus accounted for 31.3% of the reported comorbidities, followed by hypertension (24.1%). We found after study of the data that only 347 individuals (0.34 percent of those covered under Amritham) tested positive for the SARS-CoV-2 infection during their quarantine course under the Amritham program. All of them recovered fully without any serious complications, none of them required intensive care or ventilator support. There was no reported incident of adverse drug reactions arising from Ayurvedic medicines. Meanwhile, data collected from authentic government sources showed that 1.61 percent of the general quarantined population tested positive for Covid-19 during the corresponding period.

**Table 2:** Percentage distribution of age among quarantined individuals under Amritham (*n* = 101,218).

**Age**	**Number of individuals**	**Percentage (%)**
0 to 4	3,026	3.0
5 to 14	5,883	5.8
15 to 24	12,996	12.8
25 to 34	28,953	28.6
35 to 44	22,163	21.9
45 to 54	16,789	16.6
55 to 64	8,819	8.7
65 to 74	2,028	2.0
75 to 84	483	<1
85+	78	<1
**Total**	**101,218**	**100.0**

**Table 3:** Gender, domicile, travel, quarantine facility and comorbidity patterns of quarantined individuals under Amritham from 21 May 2020 to 8 July 2020.

**Particulars**	**Number of Individuals**	**Percentage (%)**
**Gender (*****n*** **=** **101,218)**		
Male	73,868	73
Female	27,347	27
LGBTQ	3	<1
**Reason for Quarantine (*****n*** **=** **101,218)**		
Travel	98,537	97.4
Primary Contact	2,588	2.6
Secondary	93	<1
**Travel origin (*****n*** **=** **98,537)**		
Out of India	62,207	63.1
Out of Kerala	36,330	36.9
**Principal travel mode (*****n*** **=** **98,537)**		
Air	60,968	61.9
Road	27,400	27.8
Rail	10,148	10.3
Water	21	<1
**Allotted quarantine facility (*****n*** **=** **101,218)**		
Home quarantine	93,316	92.2
Institutional quarantine	7,902	7.8
**Pattern of comorbidity (n** **=** **101,218)**		
with comorbidities	19,473	19.2
without comorbidities	81,745	80.8
**Gender-wise distribution of comorbidities (*****n*** **=** **19,473)**		
Male	14,119	72.5
Female	5,354	27.5
LGBTQ	0	0

## Discussion

Ayurveda, a legitimized and well-accepted traditional medical system in India is integrated into the health system in Kerala through an extensive network of primary, secondary and tertiary institutions. Despite the integrated network, problems such as inadequate documentation and reporting of the clinical and public health outcomes have been the impediments towards building an evidence-based policy approach in Ayurvedic protocols, hitherto. The government has started focusing on building scientific and evidence-based data systems to assess the efficacy of promotive, prophylactic and therapeutic protocols of Ayurveda in various disease conditions.

The Ayush Department worked out a decentralized, people-centered and participatory organizational framework for implementing the Ayurvedic preventive and therapeutic strategies during the COVID-19 pandemic, with a view to strengthening the reach, penetration, sustainability, and scalability of Ayurvedic services and for establishing robust reporting mechanisms. The current approach in the Kerala setting seems novel and does not seem to have been attempted in other Indian states.

We have found that such a systematized approach facilitated the delivery of Ayurvedic medicines to Covid-19 quarantined individuals and facilitated the collection and dissemination of data. The Amritham program reached out to 25.7% of quarantined individuals in Kerala from 21 May 2020 to 8 July 2020. Further, only 0.34% of these individuals tested positive for SARS-CoV-2 infection. These individuals later recovered without developing any severe complications or Covid sequelae. However, a detailed analysis has not been done to assess the impact of other co-morbidities and pre-existing health conditions on the general health outcome of quarantined individuals.

Government of India (GOI) approved the use of Ayurvedic regimen in boosting the immunity of the population against COVID-19 in the initial stage of the pandemic itself (45) Government of Kerala considered the main highlights of the GOI strategy while articulating the strategy of the state. Nevertheless, the data that has emanated from the systematic implementation of the strategy in the state needs thorough and rigorous scientific analysis and review for widening the scope of the strategy in other public health emergencies. Recent literature has shown that systematic and integrative use of traditional medicine and conventional medicine offered better clinical outcomes in China with no adverse drug reactions and prevented deterioration of the disease (46–48). Similar findings are also available from India (49, 50). In-silico and preclinical studies on several medicinal plants included in Ayurvedic protocols against various stages of SARS-CoV-2 infection also yielded significant results (51, 52). Furthermore, attempts to establish clinical utility of Ayurvedic formulations are also reflected in the clinical trial registry of India in which a considerable proportion of trials had registered with Ayurvedic drugs in COVID-19 (53, 54).

The 1,206 life clinics cover all the 941villages with a jurisdiction of over six million households across Kerala (55). These clinics have been functioning adequately in tandem with the existing conventional medicine-oriented public health machinery. As of 28 June 2021, over 3.1 million people in Kerala received the benefits of different Ayurvedic programs for the pandemic (Table 1). The continued delivery of services indicates the sustainability of the present policy-based implementation framework.

There were seasonal epidemic outbreaks, earlier too, in Kerala, such as dengue and chikungunya (56, 57). Although the public Ayurveda machinery in the state had supported mitigatory effects of the government during such epidemic outbreaks, the absence of an organized approach, sparse documentation, and lack of publication reduced the scalability of the adopted Ayurvedic interventions. Conversely, the data collection mechanism under the State Ayurveda COVID-19 Response cell has improved the scalability of various Ayurvedic interventions for the pandemic in the current scenario. The approach could also stream-line the daily reporting of COVID-19 and collection of critical data regarding the effectiveness of the Ayurvedic programs in different pandemic stages, including adverse drug reactions from Ayurvedic medicines (none reported).

We overcame the scarcity of skilled human resources and infrastructure for service delivery and data-collection through a public-private-participatory model, with active involvement of the LSG bodies and local community. The method also enhanced the awareness of the general public about various Ayurvedic programs available for COVID-19. Apart from providing substantial financial assistance, the support from the LSG bodies helped the department overcome several operational hurdles in reaching the targeted population and in delivering medicines. The awareness generated through social media created social linkages necessary for pandemic response. Coordination between various government departments and professional organizations was crucial to improving communication and subsequent stakeholders' involvement.

Globally there is convincing data regarding positive collaboration between health- care and non-healthcare organizations in improving public health (58, 59). Our experience has shown that in the present context, collaboration with the local self-government systems, voluntary organizations, health workers, and public health machinery considerably improved the delivery of Ayurvedic services in Kerala during the initial pandemic wave. With a dynamic decentralized governance system having been built over the years, and active involvement of the general public at the grass-roots level, Kerala has been able to effectively manage earlier crises arising out of natural calamities and even the Nipah outbreak of 2018 and 2019 (28, 60). Additionally, proper communication strategy, integration of technology with public health-care delivery and active community involvement have contributed to the successful operationalization of the Ayurvedic strategies for mitigating the impact of COVID-19 pandemic in the state (30).

We have identified few lacunae in the implementation of the framework. Lack of prior experience in handling a public health crisis of such magnitude through an integrated multi-sectoral approach, lockdowns and subsequent closure of academic institutions were the critical hurdles in the implementation. The other identified obstacles were the scarcity of skilled human resources in epidemiological and field-level activities and lack of adequate infrastructure for precise delivery of medicines and for collection and dissemination of data. We have relied on secondary data from authentic government sources to gather information on the Covid positivity of quarantined individuals. Further, extensive data gathered from the field related to Amritham and its consolidation might have attracted various biases. The program implementation has continued with an ongoing in-depth analysis of the gathered data, which would help identify and resolve some of these biases. Succinctly, the single most barrier to the development, implementation and dissemination of Ayurvedic strategies for Covid was the lack of resources. The prominent facilitators to the strategy were the support from the local self-governance system, active involvement of the Ayurvedic private sector and social participation.

Altogether, the lessons learnt from the reported community interventions are as follows;

1. Policy-oriented, decentralized and well-planned implementation processes can improve the reach, penetration, scalability and sustainability of services even in resource-poor settings in an epidemic.
2. Kerala's decentralized governance, social democracy and social participation can facilitate robust community-level activities.
3. An implementation system with social participation can create awareness about services and improve the system's functioning.
4. The scalability of implemented programs and constant reporting of community interventions during a pandemic can bring sustainability in public health activities and aid formulation of new policies.
5. Rather than promoting a pluralistic approach, an integrated approach to public health delivery where Ayurveda/traditional medicine systems' services can be provided to the public with the collaboration of the local self-governments may be a better alternative.

Broadly, the findings of this community study can work as a model for developing a well-organized integrative health system and as reference material for building a stronger science-policy interface. The model can also help governments mainstream evidence-based traditional medicine components to existing public health strategies and practices for better outcomes. A detailed understanding and critical analysis of such models and their elements can enrich traditional medicine and public health education and research at large. We recommend that national and state governments adopt the health system model we have reported for facilitating the delivery of traditional medicine services in resource-poor settings. While doing this, good data collection, monitoring, and reporting mechanisms are indispensable for timely evaluation of the mechanisms in place and for bringing about necessary corrective measures to the system. Identification of critical personnel with well-defined duties and responsibilities coupled with standard operating procedures will enable smooth functioning of the system. Regular discussions among various stakeholders and responsible persons can help build trust. Various capacity building and training programs are essential for delivering services at the grassroots and for the proper functioning of the operational framework. Rigorously designed, ready-to-implement health system research strategies are necessary for creating sustainable public health management models in the traditional medicine sector. Therefore, we also recommend extensive translational research in Ayurveda that can pave the way for developing clinically viable products for community use. At the same time, exploring valuable clinical and therapeutic information from Ayurvedic treatises is also required for integrating Ayurveda into the existing public health system.

## Conclusion

Since the evolution of the COVID-19 pandemic, we have focused on the synthesis of existing practice-based evidence and the development of evidence-based practice. The Government of Kerala worked out a feasible decentralized and participatory framework model for delivering and reporting Ayurvedic services replicable in resource-poor settings. Better utilization of these services can occur with an integrative health system approach. The establishment of accurate scientific evidence on the efficacy of the Ayurvedic management protocols would accelerate such integration and mainstreaming of Ayurveda.

## Data Availability Statement

The original contributions presented in the study are included in the article/Supplementary Material, further inquiries can be directed to the corresponding author/s.

## Ethics Statement

Ethical review and approval was not required for the study on human participants in accordance with the local legislation and institutional requirements. Written informed consent for participation was not required for this study in accordance with the national legislation and the institutional requirements.

## Author Contributions

RP created the initial draft manuscript. SJ and DI edited and developed the manuscript. All authors contributed equally to the research and submission process.

## Conflict of Interest

The authors declare that the research was conducted in the absence of any commercial or financial relationships that could be construed as a potential conflict of interest.

## Publisher's Note

All claims expressed in this article are solely those of the authors and do not necessarily represent those of their affiliated organizations, or those of the publisher, the editors and the reviewers. Any product that may be evaluated in this article, or claim that may be made by its manufacturer, is not guaranteed or endorsed by the publisher.
