# Supplementary material for: Ayurvedic Response to COVID-19 Pandemic in Kerala, India and Its Impact on Quarantined Individuals – A Community Case Study
Source: Front Public Health. 2021 Oct 15;9:732523. doi: 10.3389/fpubh.2021.732523 (PMC8554199; doi:10.3389/fpubh.2021.732523)
Supplement: Supplementary file 1 [file Table_1.docx]

**Figure 2 dataset**

Table1: District-wise percentage of COVID-19 quarantined individuals enrolled in Amritham from 21May 2020 to 8 July 2020 in Kerala.

| **District** | **Total number of individuals quarantined^*^** | **Individuals quarantined under Amritham** | **Percentage (%)** |
| --- | --- | --- | --- |
| Ernakulam | 36888 | 4123 | 11.2 |
| Thiruvananthapuram | 37922 | 5163 | 13.6 |
| Kannur | 33876 | 4652 | 13.7 |
| Idukki | 17674 | 2509 | 14.2 |
| Kasaragod | 13576 | 2076 | 15.3 |
| Pathanamthitta | 19283 | 3452 | 17.9 |
| Kozhikode | 47528 | 8734 | 18.4 |
| Kottayam | 19383 | 4386 | 22.6 |
| Wayanad | 14866 | 3707 | 24.9 |
| Kollam | 26648 | 8935 | 33.5 |
| Malappuram | 50374 | 18201 | 36.1 |
| Alappuzha | 19840 | 7530 | 38.0 |
| Thrissur | 35259 | 13533 | 38.4 |
| Palakkad | 27554 | 14217 | 51.6 |

*^*^Total quarantine data for the period sourced from the official web portal of the Kerala Government available at https://covid19jagratha.kerala.nic.in/home/addSurveillanceDashboard, accessed on 10 July 2020.*

Table 2: Statewide percentage of COVID-19 quarantined individuals enrolled in Amritham from 21May 2020 to 8 July 2020 in Kerala

|  | **Total number of individuals quarantined^*^** | **Individuals quarantined under Amritham** | **Percentage (%)** |
| --- | --- | --- | --- |
| Kerala^#^ | 394269 | 101218 | 25.7 |

*^*^Total quarantine data for the period sourced from the official web portal of the Kerala Government available at https://covid19jagratha.kerala.nic.in/home/addSurveillanceDashboard, accessed on 10 July 2020.*

*^#^The state data represents the verified and finalized quarantine data from the districts for the period, which was slightly less than the cumulative district quarantine data.*
